# Case of Amputation above the Shoulder Joint

**Published:** 1854-10

**Authors:** D. Gilbert

**Affiliations:** Professor, &c. Med. Dept. of Penna. College


*Case of Amputation above the Shoulder Joint.* By D. Gilbert, M. D., Professor, &c. Med. Dept. of Penna. College.

I was requested to visit, on the 29th of June, 1854, David Thompson, æt. 24 years, carpenter, residing on 19th St. above Fairview in this city. Saw him for the first time two days subsequently, June 1st, when he gave me the following history of his case. About the 1st of January last he slipped and fell on the ice, and to break the fall, threw his right arm back and fell upon his hand, which resulted in severe sprain of the shoulder joint. There being neither fracture nor dislocation, he did not apply for medical advice, but made such applications as are common in domestic practice. During this period he continued to work at his trade, without, however, being able to use the shoulder joint freely. Motion at the joint becoming more and more abridged and painful, he applied to a surgeon for advice about seven weeks after the receipt of the injury. At this time there was considerable tumefaction of the shoulder. His medical adviser requested him to keep the arm at rest, and used counter-irritants to the tumor, and gave sorbefacients and other remedies internally. The swelling, however, gradually increased, the shoulder became more painful, when motion of the joint was attempted, and during the night a dull heavy pain was experienced even when at perfect rest.

I made a careful examination, and found a large globular tumor involving the ends of all the bones forming the joint, extending as low down as the insertion of the deltoid muscle, encircling the humerus, obliterating the axillary cavity, and resting upon the chest opposite to the articulation. The surface of the tumor was evenly rounded and free from nodulation, the integument covering it was unattached and normal in appearance, except a little abrasion of the cuticle and discoloration produced by the counter-irritants used. The body of the tumor was firmly elastic, without the least fluctuation, as if homogeneous in structure. There was some tenderness on pressure, especially anterior to the acromion where the principal part of the injury was sustained. By measurement, the circumference of the tumor, taking the axilla and acromion as points, was found to be 19½ inches, whilst the sound shoulder measured 13½ inches. The horizontal arc of the body of the tumor from the chest anteriorly to the chest posteriorly was 14½ inches; on the sound side 9 inches. A line coinciding with the axis of the lower two thirds of the shaft of the humerus continued upwards through the tumor would have emerged, as near as we could judge, about one and a half inches anterior to the acromion process, showing that the upper third of the humerus which was involved in the tumor had undergone change of form. In tracing the bone up into the tumor, increase in its circumference was clearly evident, and then it became blended with the general mass. When the tumor was steadied with one hand, the lower extremity of the humerus could be moved in all directions, so as to cause the line of its axis at the top of the shoulder, to describe a circumference whose diameter was at least six inches, having the acromion as its centre.

The patient had been, up to this time, able to sit up and even pass from room to room. Since the receipt of the injury, there has been a gradual wasting of the fluids and solids of body, his weight having become reduced from one hundred and seventy to one hundred and thirty-three pounds. His complexion is somewhat inclined to sallowness, but by no means cachectic; his pulse is one hundred and twelve, and quick, appetite and digestion variable, and his alvine evacuations are irregular. There has not been any cough, and the physical signs declare the thoracic organs to be in a healthy condition. Prior to this accident, from his earliest years he enjoyed uniform and uninterrupted good health, and never had a single symptom of scrofulous or any other constitutional disease. Mr. T. is the youngest of thirteen children, none of whom had struma or any form of cachectic disease. Six of these are dead, four died of small pox, one of enteritis, and one of hæmorrhage of the lungs. His father is still living at the advanced age of 76. His mother died it was said, of carcinoma uteri at the age of sixty-three, when the patient was seventeen years old. All his ancestors lived to a great age. His maternal grandmother died recently at the age of over one hundred years. I ordered laxative pills to be taken at bed time, and syrup sarsap. comp. with iodid. potass., in ordinary doses three times a day. Locally tinct. arnica montan., which had been previously used.

June 3d.—Visited patient; find no change worthy of note; sleeps tolerably well, no pain in the shoulder when quiet; bowels were opened by the pills; appetite has improved. Continue treatment.

June 8th.—Pulse 108; bowels have required an occasional pill; appetite continues to be moderately good; fore arm begins to be œdematous; skin over tumor is becoming brawny, in spots as large as a silver dollar; emaciation is evidently progressing; sleep disturbed; irritative fever at night. Continue treatment with grs. v. of Dover's powder at bed time.

June 11th.—Symptoms very much the same; tumefaction increasing; measurement to day $21\frac{1}{2}$ and $16\frac{1}{2}$ inches; brawny spots enlarging; no adhesion, however, between the skin and tumor. At my request, my colleague, Dr. John Neill, was called in consultation.

June 12th.—Met Dr. Neill; found increase of the unfavorable symptoms; pulse 126; œdema of arm and tumor increasing; general emaciation progressing. Continue treatment.

June 13th.—No material change. Treatment continued.

14th.—Saw the patient in company with Dr. Neill. Finding that the case was progressing steadily towards a fatal termination, there being an increase of all the unfavorable symptoms, the propriety of performing the operation of amputation above the shoulder-joint was considered. It was agreed that the operation afforded the only hope of relief; but the decision, as to the propriety of its performance, was postponed.

I visited the patient daily, found an aggravation of the symptoms, to which severe nasal hemorrhage was added; pulse 132 to 140; night sweats.

18th.—Met Dr. Neill; unfavorable symptoms progressing, softening of tumor commenced. It was agreed, after mature consideration, that the amputation ought to be performed, inasmuch as it afforded the only hope of rescuing the patient from impending dissolution, provided he and his friends desired it, after a full statement of the dangers attendant on its performance; and the doubtful issues involved, even if he should survive the immediate effects of the operation.

19th.—Visited the patient, and made my communication to him. He had anticipated its character, and was prepared to give a very decided answer in favor of the operation. His friends united with him in the urgent request that it should be performed; and consequently, Thursday, the 22d, at 4 o'clock, P. M., was appointed for the operation.

Measurement of the tumor 24 and 19 inches; œdema of fore arm increased.

All necessary preparation having been made, the operation was accordingly performed at the hour appointed, in the presence of Drs. J. Neill, B. Henry, J. M. Allen, W. H. Gobrecht, E. B. Jackson, W. K. Gilbert, F. C. Jaquet, and a number of medical students, in the following manner:—

The patient was placed upon his left side, on a firm table six feet long and two feet wide, provided for the purpose and prepared with the necessary coverings. Dr. Gobrecht using Dr. Bond's instrument for *retroversio uteri* instead of a key, compressed the sub-clavian artery; for which purpose this instrument, on account of its long bent stem, is admirably adapted. Dr. Neill took charge of the arm to be removed, and I took my place above, or rather behind the head of the patient. A mixture of one part of chloroform to three of ether was administered. The operation was commenced by an incision made with a large scalpel, commencing at a point below the middle of the clavicle, and continued forward and downward to a point below the acromion; thence backward and upward, and outside of the spinous process of the scapula, to the middle of this process. The triangular flap thus formed was dissected up; an incision was now commenced at the posterior fold of the axilla, and carried up to the place of termination of the first incision. The muscles under the line of the last incision, as well as the supra and infra spinati muscles, which were laid bare by the dissection of the flap, were now all divided. The amputating saw was applied to the spinous process of the scapula, and this portion of the bone was sawn through obliquely, downwards and forwards to the body of the scapula; and this was next divided above its neck; the clavicle was then isolated at its central point, and cut through with Hey's saw. A middle sized catling was introduced into the posterior incision, and rapidly carried under the coracoid process, and brought out between the divided ends of the clavicle, severing in its course, the sub-scapularis, pectoralis minor and major muscles, blood vessels, nerves and integuments, so as to form the anterior flap; and the amputation was completed.

The subclavian artery was immediately secured by a firm ligature; but so perfect was the compression of this vessel, that no blood was lost by it. Six other enlarged arterial trunks required ligatures. The remaining portion of the clavicle and scapula were approximated; and the upper triangular, and anterior and posterior flaps were brought together over these, and secured by nine sutures, and intervening adhesive plasters; lint wetted with cold water was applied, and the patient carried to bed. The anæsthesia was complete, and so successfully kept up, that the patient was totally insensible to suffering. Only about twenty oz. of blood, principally venous, was lost. Pulse immediately after the operation, was 120; but in two hours after, had increased to 140. Stimulants, anodynes and fluid nourishment having been administered, it again came down to 122; and tranquility, with sleep, took the place of the restlessness which was present soon after the operation.

I might here add the notes which were taken during the after treatment, for the first day, every hour; and subsequently, from four to six times every twenty-four hours, detailing minutely the symptoms and treatment of this case, but they would occupy a large space and add very little that is interesting. There seemed to be a constant tendency to sinking of the vital powers, which in the treatment demanded the free use of stimulants, tonics, anodynes, and a nutritious and easily digested diet. Thus the contest was maintained for a period of eight days after the operation was performed, when the patient sank quietly in death, requesting near the close of life that the attending physicians should be assured that the operation and its effects had been entirely painless, and that he rejoiced that this chance for life had been given to him. A post-mortem examination was not permitted.

The following account of the dissection of the tumor has been kindly furnished by Dr. Neill.

A section of the shoulder, through to the bone, revealed those changes which are consequent upon the growth of a medullary cancer. The deltoid was thin and pallid, and tightly stretched over the tumor, like a fascia; all the tissues exhibited the effects of pressure, distension and infiltration.

The tumor was of that form, which Paget characterizes as soft medullary cancer. The interior was composed of a pulpy, brain-like material; rendered somewhat of a pink color by its great vascularity. The deeper portion, that nearest the bone, was softened almost to fluidity. The head and neck of the humerus were entirely absorbed, and loose fragments, very much eroded, were lying in contact with the upper end of the shaft. The microscopic characters were such as might have been anticipated, with such physical conditions. The cells were very large and numerous, and so filled with oil globules, that the nuclei were often obscured.

*Remarks.*—This case is in all respects, similar to that of the Hon. J. Wagonseller, M. D., late of Selings Grove, Pa., reported in the American Journal of the Medical Science for

Oct. 1847. It is somewhat singular that these the only cases, in which the *operation of amputation above the shoulder joint* has been performed, occurred in the practice of the same individual. I take it for granted, that most surgeons would have had recourse to the same procedure. In both these cases, the advice and full concurrence of eminent surgeons, as to the propriety and necessity of the operations, was given in consultation. In addition to this, numerous medical friends, occupying high positions in the profession, gave to them their unhesitating sanction, prior and subsequent to their performance.

In comparing these cases we find that the exciting cause was the same in both, viz., mechanical injury sustained by the joint in falling. The progress of the disease was more rapid in the last than in the first case. This probably was owing to the difference in their respective ages and previous states of health, which had been impaired for years in the first, while in the latter case it had been unimpaired. All the symptoms considered, the local disease presented more decided characteristics of malignancy in the case of Mr. Thompson, whilst the evidences of constitutional dyscracy were more strongly marked in the case of Dr. Wagonseller. The operation found more reactive power and elasticity of vital vigor in the constitution of the latter, which had been inured to suffering for years, than in the former, which had always been healthy.

In the performance of the operation, the division of the clavicle, in the last case, was deferred until after the spinous process and body of the scapula were sawed through. This greatly facilitated the most difficult steps of the operation, since the diseased mass and shoulder were held clear of the thorax. The time occupied in the performance of the first operation was fourteen minutes; in the last eight minutes. This difference in time, which is important in amputations in general, may be attributed mainly to the anæsthesia, and furnishes another instance of the great value of this discovery to surgery. When my first operation was performed, the anæsthetic agent then in partial use had not yet been adopted by the profession in this city, on account of the secrecy which was maintained in reference to its chemical composition. At the suggestion of the late Prof. Geo. McClellan, who was associated with me in the case, I visited the office of a respectable dentist of this city and witnessed its effects when teeth were extracted for two young ladies. The insensibility was perfect, but I observed that under the influence of the "*letheon*" the pulse of one rose from the normal standard to 120, and of the other to 130 beats in the minute. This directly stimulating effect, together with the charlatanism which characterised it, influenced me at once not to use it. Soon afterwards, however, it was published to the profession that "*letheon*" consisted of washed sulphuric ether, and then it was introduced into general use. During the winter of 1847–48 the discovery of *chloroform* by Dr. Simpson of Edinburgh was announced, and this soon became extensively introduced as preferable to ether.

The writer of this used chloroform first in the extraction of two teeth before the class of the Medical Department of Pennsylvania College on the 7th of January, 1848, and next day in the amputation of a female mamma in Kensington. The anæsthesia was complete, but a depressing or sedative influence was noticed, and it became necessary to administer ether to restore normal action. Observing this lowering effect of chloroform in other cases, and knowing well that the effect of ether was restorative, I came to the conclusion that if these two agents could be mingled before their exhibition without deterioration or alteration in their anæsthetic properties, great advantage would be the result. I consequently consulted my learned friend M. Jacobs, Professor of Chemistry and Natural Philosophy in the Pennsylvania College at Gettysburg, who at once assured me that in such mixture no change of their original properties would be produced. Consequently in my next operation, which was performed Feb. 10th, 1848, for the removal of a large adipose tumor from the neck of a lady, I used a mixture consisting of four parts, by bulk, of sulph. ether, and one of chloroform, with the happiest results. Since then, in all surgical and other cases in which anæsthesia was required, I have used the mixture of these two agents. The proportions of these, I have varied according to the supposed vital constitutional vigor present in the patient to be operated upon. In the robust, the proportion of chloroform was increased; in the enfeebled, the quantity of ether greatly preponderated. I append this brief history of my use of these anæsthetic agents, because my first case of amputation above the shoulder-joint, gave rise to my earliest investigations upon the subject of anæsthesia; and this, subsequently, led to the first use of the mixture of the two agents, which I freely communicated to my professional brethren, and has since been extensively introduced.

